# Targeting p62 by sulforaphane promotes autolysosomal degradation of SLC7A11, inducing ferroptosis for osteosarcoma treatment

**DOI:** 10.1016/j.redox.2024.103460

**Published:** 2024-12-05

**Authors:** Qiuming Zou, Xiaofeng Zhou, Jianqin Lai, Haixia Zhou, Jinxuan Su, Zhijing Zhang, Xiaosong Zhuang, Lili Liu, Ruijie Yuan, Sijia Li, Siyu Yang, Xinyi Qu, Jiezhu Feng, Yongqi Liu, Zisheng Li, Shiting Huang, Zhi Shi, Yu Yan, Zhiming Zheng, Wencai Ye, Qi Qi

**Affiliations:** a State Key Laboratory of Bioactive Molecules and Druggability Assessment, MOE Key Laboratory of Tumor Molecular Biology, Guangdong Province Key Laboratory of Pharmacodynamic Constituents of TCM and New Drugs Research, Department of Pharmacology, School of Medicine, Jinan University, Guangzhou, 510632, China; b Department of Gastrointestinal Surgery, Guangzhou First People's Hospital, Guangzhou, 510180, China; c State Key Laboratory of Bioactive Molecules and Druggability Assessment, Center for Bioactive Natural Molecules and Innovative Drugs Research, Guangdong Basic Research Center of Excellence for Natural Bioactive Molecules and Discovery of Innovative Drugs, Guangdong Province Key Laboratory of Pharmacodynamic Constituents of TCM and New Drugs Research, College of Pharmacy, Jinan University, Guangzhou, 510632, China; d School of Medicine, South China University of Technology, Guangzhou, 510006, China; e Department of Cell Biology & Institute of Biomedicine, Guangdong Provincial Biotechnology & Engineering Technology Research Center, Guangdong Provincial Key Laboratory of Bioengineering Medicine, Genomic Medicine Engineering Research Center of Ministry of Education, MOE Key Laboratory of Tumor Molecular Biology, National Engineering Research Center of Genetic Medicine, State Key Laboratory of Bioactive Molecules and Druggability Assessment, College of Life Science and Technology, Jinan University, Guangzhou, 510632, China; f Functional Experimental Teaching Center, School of Medicine, Jinan University, Guangzhou, 510632, China; g Department of Neurosurgery, Shandong Provincial Hospital Affiliated to Shandong First Medical University, Jinan, 250021, China

**Keywords:** Ferroptosis, Sulforaphane, p62, SLC7A11, Osteosarcoma

## Abstract

Osteosarcoma (OS) is the most prevalent malignant bone tumor in children and adolescents worldwide. Identification of novel therapeutic targets and development of targeted drugs are one of the most feasible strategies for OS treatment. Ferroptosis, a recently discovered mode of programmed cell death, has been implicated as a potential strategy for cancer therapy. Sulforaphane (SFN), the main bioactive compound derived from cruciferous vegetables, has shown potential anti-cancer effects with negligible toxicity. However, the role of ferroptosis in the effect of SFN on OS remains unknown. In the present study, we found that SFN acted as a potent ferroptosis inducer in OS, which was demonstrated by various inhibitors of cell death. The SFN-induced ferroptotic cell death was characterized by elevated ROS levels, lipid peroxidation, and GSH depletion, which was dependent on decreased levels of SLC7A11. Mechanically, SFN directly targeted p62 protein and enhanced p62/SLC7A11 protein-protein interaction, thereby promoting the lysosomal degradation of SLC7A11 and triggering ferroptosis. Notably, both subcutaneous and intratibial OS models in nude mice confirmed the ferroptosis associated anti-cancer efficacy of SFN *in vivo*. Hence, our findings demonstrate that SFN exerts its anti-cancer effects through inducing SLC7A11-dependent ferroptosis in OS, providing compelling evidence for the application of SFN in OS treatment.

## Graphical abstract

**Figure:**
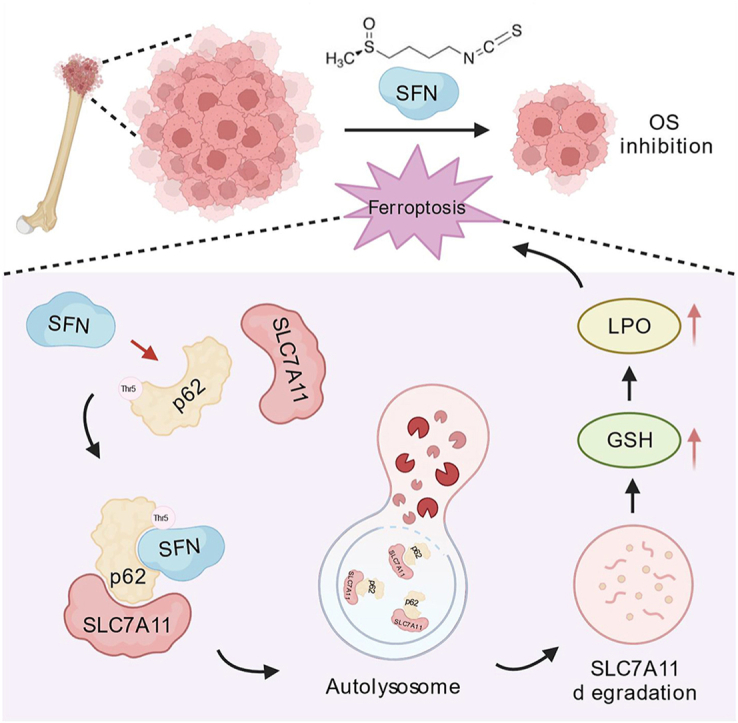

## 1 Introduction

Osteosarcoma (OS), accounting for approximately 56 % of bone sarcomas, is the most malignant bone tumor, primarily afflicting children, adolescents, and young adults, with a median age of 16 years [1,2]. The current standard of care for OS includes extensive surgical resection, neoadjuvant chemotherapy, and adjuvant chemotherapy [3], which has improved survival rates and increased the potential for limb preservation. However, the long-term administration of chemotherapy drugs takes a significant toll on normal tissues, particularly the heart, liver and kidneys [4]. Additionally, OS has a strong tendency for local invasion and early metastasis with a recurrent rate of 30∼50 % [5]. Therefore, it is crucial to identify new therapeutic targets and agents for the treatment of OS.

**Fig. 1:**
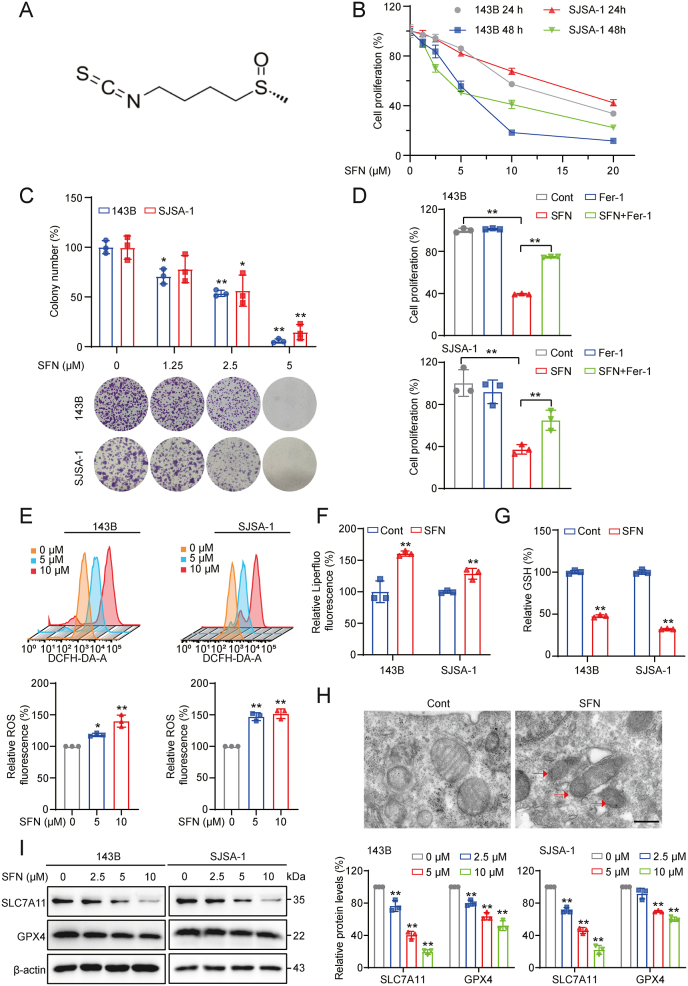
**Ferroptosis contributes to SFN-induced cell death in OS cells. (A)** Chemical structure of sulforaphane (SFN). **(B)** 143B and SJSA-1 cells were subjected to varying concentrations of SFN for 24 or 48 h, and their viability was evaluated using the MTT assay. **(C)** Representative images of the colony formation and quantitative analysis. **(D)** 143B and SJSA-1 cells were treated with SFN (5 μM or 10 μM) with or without Fer-1 (5 μM) for 48 h, and cell viability was measured. **(E)** 143B and SJSA-1 cells were exposed to SFN for 12 h, followed by the analysis of ROS levels using a flow cytometer. **(F)** Lipid peroxidation levels were assessed using a flow cytometer after 143B and SJSA-1 cells were treated with SFN (10 μM) for 12 h. **(G)** 143B and SJSA-1 cells were treated with SFN (10 μM) for 12 h, and GSH levels were measured. **(H)** Transmission electronic microscopy was used to observe the ultrastructure of 143B cells. Cells in the SFN (10 μM, 12 h) group displayed mitochondrial shrinkage, increased mitochondrial membrane density, and reduced mitochondrial cristae. Red arrow: mitochondrial change in SFN-treated cells. Scale bar: 500 nm. **(I)** Western blotting was performed to detect the expression of ferroptosis-related proteins in osteosarcoma cells following SFN treatment. ∗*P* < 0.05, ∗∗*P* < 0.01.

Sulforaphane (SFN, Fig. 1A), an isothiocyanate derived from cruciferous vegetables, particularly broccoli sprouts [6], has been revealed for its anti-tumor potential in various types of cancers with negligible toxicity [[7], [8], [9]], which is achieved through several mechanisms, such as inhibiting phase I enzymes that activate carcinogens, inducing nuclear factor erythroid 2-related factor 2 (Nrf2)-regulated genes of phase II detoxification enzymes, and triggering apoptotic cell death [6,[10], [11], [12], [13], [14], [15]]. Additionally, SFN has been found to suppress angiogenesis by regulating vascular endothelial growth factor and matrix metalloproteinase-2 [16], as well as inhibiting metastasis [17,18]. Notably, SFN also can pass the blood-brain barrier and exert therapeutic effects against neurodegenerative diseases [19,20]. However, the specific anti-cancer effects and mechanisms of SFN against OS remain incompletely understood.

Ferroptosis is a novel form of iron and ROS-dependent cell death distinct from cell death induced by autophagy and apoptosis [21], which is characterized by cytological changes, including reduced or absent mitochondria cristae, a ruptured outer mitochondrial membrane, and a condensed mitochondrial membrane [[22], [23], [24], [25], [26]]. The dysregulation of ferroptosis has been closely associated with various human cancers [27,28]. Accumulating evidence highlights the pivotal role of ferroptosis in tumor suppression and considers its induction as a new therapeutic strategy for cancer treatment [[29], [30], [31]]. However, the role of ferroptosis and its application in OS treatment remain poorly understood.

It has been revealed that ferroptosis is regulated by several key factors, including SLC7A11 and GPX4 [[32], [33], [34], [35]]. SLC7A11, located on the cell membrane, imports extracellular cystine into the cell, which is subsequently converted to GSH [36]. GPX4 utilizes GSH to reduce lipid hydroperoxides, thus inhibiting ferroptosis [32]. As such, SLC7A11 acts as a suppressor of ferroptosis, and their inhibition can trigger ferroptosis [21,32]. The expression and activity of SLC7A11 are regulated at multiple levels, encompassing both translational and posttranslational mechanisms. For translational regulation, SLC7A11 can be induced by Nrf2 and regulated by BAP1 and the methylation of histone H3 [29,37,38]. Posttranslational regulation of SLC7A11 primarily involves the lysosomal system and proteasome pathway, which act synergistically or independently to maintain intracellular protein homeostasis, thereby ensuring the selective degradation of biomaterials in cells [39]. Notably, OTUB1, a noncanonical deubiquitinase, has been shown to interact with SLC7A11 to prevent its degradation [40]. The adhesion molecule CD44 variant (CD44v) serves as a binding partner, modulating SLC7A11 protein stability [41]. However, the specific mechanisms and regulation of SLC7A11 in ferroptosis in OS remain largely unexplored.

In the present study, we found that SFN acts as a potent ferroptosis inducer in OS and significantly blocks OS growth *in vitro* and *in vivo*. Further data indicated that SFN directly targets p62 protein and promotes the lysosomal degradation pathway of SLC7A11 through enhancing p62/SLC7A11 protein-protein interaction. These findings provide compelling evidence that targeting SLC7A11 is a promising strategy for OS treatment and lay the foundation for the application of SFN in OS therapy.

## 2 Materials and methods

### 2.1 Cell culture

Human OS cells (143B and SJSA-1) and HEK293T cells were obtained from the American Type Culture Collection (ATCC). 143B cells were cultured in MEM medium supplemented with 10 % fetal bovine serum (FBS) and 1 % penicillin-streptomycin (PS). SJSA-1 cells were cultured in RPMI-1640 medium with 10 % FBS and 1 % PS. HEK293T cells were cultured in Dulbecco's Modified Eagle Medium (DMEM) supplemented with 10 % FBS and 1 % PS. All cells were maintained at 37 °C in a humidified incubator containing 5 % CO_2_.

### 2.2 Animal study

All animal experiments were conducted in compliance with the protocols approved by the Use and Care of Animals Committee at Jinan University. A subcutaneous tumor-bearing nude mouse model was established by injecting 5 × 10^6^ 143B cells into the flank of BALB/c nude mice. Six days post-injection, mice were randomly assigned to one of three groups and received intraperitoneal injection of either 200 μl of normal saline, SFN (25 mg/kg every two days), or SFN (50 mg/kg every two days). Tumor dimensions and body weight were recorded every two days following the initial drug administration. Tumor volume was calculated using the formula: (tumor long diameter × tumor short diameter^2^)/2. At the end of study, mice were euthanized, and tumors were collected for immunohistochemical (IHC) staining.

For the intratibial xenograft model, BALB/c nude mice were anesthetized with 0.5 % pentobarbital sodium (ip, 200 μl/mouse). 143B cells (4 × 10^7^ cells/ml) suspended in PBS (25 μl/mice) were orthotopically injected into the proximal tibia. When tumors reached approximately 80 mm^3^, the tumor-bearing mice were randomly assigned to receive normal saline, Fer-1 (2 mg/kg), SFN (50 mg/kg), or SFN + Fer-1 every two days. Following the treatment, mice were euthanized, and tumor tissues were collected for further analysis.

### 2.3 Reagents and antibodies

Sulforaphane (TRC-S699115) was acquired from Toronto Research Chemicals. Deferoxamine (HY-B0988), MG132 (HY-13259), Bafilomycin A1 (HY-100558), Ferrostatin-1 (HY-100579), Liproxstatin-1 (HY-12726), Necrostatin-1 (HY-15760), Z-VAD-FMK (HY-16658B), and chloroquine (HY-17589A) were purchased from MCE. Antibodies included anti-Ki67 (Servicebio, GB121141-100) and anti-GPX4 (59735), anti-SLC7A11 (12691), anti-β-actin (3700), anti-p62 (23214), anti-LC3B (3868), anti-rabbit IgG (H + L), Biotinylated Antibody (14708), and Anti-mouse IgG (H + L), Biotinylated Antibody (14709) from the Cell Signaling Technology.

### 2.4 Cell viability analysis

Cell viability was assessed using the 3-(4,5-Dimethylthiazol-2-yl)-2,5-diphenyltetrazolium bromide (MTT) assay. Cells were seeded in 96-well plates at a density of 5 × 10^3^ cells per well, cultured for 24 h, and subsequently treated with SFN. Following incubation, the cells were exposed to 5 mg/ml MTT solution at 37 °C for 2 h. Formazan was dissolved in DMSO and optical density was read in a microplate reader with a wavelength of 595 nm.

### 2.5 Colony formation assay

Cells were seeded in 6-well plates at a density of 1 × 10^3^ cells per well. The culture medium with or without drugs was replenished every 2–3 days. Colonies were fixed, stained by 0.1 % crystal violet, and colonies were quantified using ImageJ software.

### 2.6 Quantitative real-time PCR

Total RNA was extracted using TRIzol (Invitrogen, CA, USA) and reverse transcribed using the TransScript All-in-One First-Strand cDNA Synthesis SuperMix (TransGen Biotech). For the analysis of mRNA expression, PerfectStart Green qPCR SuperMix (TransGen Biotech) was employed. The primers used are listed below: SLC7A11-F: GGGCATGTCTCTGACCATCT; SLC7A11-R: TCCCAATTCAGCATAAGACAAA; β-actin-F: ACTTAGTTGCGTTACACCCTTTCT; β-actin-R: GACTGCTGTCACCTTCACCGT.

### 2.7 ROS measurement

Intracellular ROS levels were assessed using the DCFH-DA oxidative stress indicator (Beyotime, S0033) in accordance with the manufacturer's instructions. Specifically, cells were cultured in serum-free medium supplemented with DCFH-DA at 37 °C for 30 min. Subsequently, the cells were collected, and the fluorescence signals were measured by flow cytometry.

### 2.8 Analysis of lipid peroxidation

The intracellular lipid peroxidation was quantified with MDA assay. MDA levels were determined using a Lipid Peroxidation MDA Assay Kit (Beyotime, S0131). For Liperfluo staining, cells were exposed to Liperfluo (10 μM, Dojindo, Japan) for 1 h at 37 °C, followed by trypsin digestion, and fluorescence intensity was measured by flow cytometer.

### 2.9 FerroOrange staining and GSH assay

FerroOrange (Dojindo, Japan) is a fluorescent probe used for live-cell imaging of intracellular Fe^2+^ ions. Live cells were incubated with FerroOrange for 1 h, and intracellular Fe^2^⁺ levels were visualized using a confocal microscope. GSH levels were conducted using a GSH Assay Kit (Nanjing Jiancheng Bioengineering Institute, A006-2-1) in accordance with the provided instructions.

### 2.10 LysoTracker staining

Cells were incubated with DMEM containing 100 nM LysoTracker Red DND-99 (Thermo Fisher Scientific, L7528) for 30 min at 37 °C. Subsequently, the cells were collected, and the fluorescence signals were analyzed using flow cytometry.

### 2.11 Immunoprecipitation (IP) and western blotting

Cells were lysed in lysis buffer (150 mM NaCl, 0.5 % NP-40, 1 mM EDTA, 10 % glycerophosphate, 50 mM Tris-Cl, and a proteinase inhibitor cocktail). Following a 30 min incubation, the cellular lysates were separated by centrifugation at 12,000 rpm at 4 °C for 15 min. The resultant supernatant was then subjected to incubation with 20 μl of protein A/G agarose beads and the specified antibody at 4 °C overnight. After extensive washing, the beads were eluted, fractionated via SDS-polyacrylamide gel electrophoresis, and subjected to immunoblotting analysis. The immunoblots were visualized using an electrochemiluminescence imaging system (Tanon, Shanghai, China).

### 2.12 Immunofluorescence staining

Cells were seeded on glass coverslips and rinsed with PBS, followed by fixation with 3.7 % formaldehyde. After fixation, the cells were washed with PBS and permeabilized using 0.1 % Triton X-100 in PBS for 10 min. Next, a blocking buffer (5 % BSA in 0.1 % Triton/PBS) was applied for 1 h, followed by overnight incubation with specific antibodies. Subsequently, the cells were washed three times with PBS and incubated with fluorescent secondary antibodies (ThermoFisher Scientific) for 2 h. Nuclei were stained with 4’,6-diamidino-2-phenylindole (DAPI, ThermoFisher Scientific), and fluorescence was observed using a confocal microscope (Zeiss).

### 2.13 Cellular thermal shift assay (CETSA)

Cells were trypsinized and resuspended in PBS containing a protease and phosphatase inhibitor cocktail. The cell suspension was divided into eight fractions and heated at a gradient of temperatures for 3 min. Subsequently, the fractions were subjected to two cycles of freezing in liquid nitrogen and thawing at room temperature, followed by centrifugation at 12000 rpm for 20 min. The supernatant was collected and analyzed using western blot assay.

### 2.14 Molecular docking

Molecular docking was conducted using AutoDock Vina 1.0.2. The crystal structure of p62 (PDB: 4MJS) and SLC7A11 (PDB: 8A9L) were obtained from the RCSB protein data bank (https://www.rcsb.org/). The structure of sulforaphane was retrieved from the PubChem database (https://pubchem.ncbi.nlm.nih.gov/). File transformations were performed using Open Babel software. Visualization of the most suitable complexes was performed using Discovery Studio 2021 Client and Pymol 2.2.0.

### 2.15 Microscale thermophoresis (MST)

HEK293T cells were transfected with a p62-mCherry plasmid, after which the protein lysates were collected using RIPA lysis buffer. Subsequently, SFN was serially diluted with PBS to concentrations ranging from 50,000–1.53 nM. The protein lysates were then treated with the SFN solutions for 5 min. The resulting mixtures were loaded into capillaries and subjected to MST measurements using a Monolith NT.115 instrument (Nanotemper, Munich, Germany).

### 2.16 Isothermal titration calorimetry (ITC)

ITC measurements were conducted using a MicroCal PEAQ-ITC instrument (Malvern) at 25 °C in a buffer containing 50 mM HEPES (pH 7.5), 150 mM NaCl, and 2 mM DTT. SFN was gradually injected from the titration syringe into the p62 protein solution, with injections spaced 120 s apart and stirring at 750 rpm. The thermodynamic response was recorded in high-feedback mode, and the raw data were analyzed using the MicroCal PEAQ-ITC software.

### 2.17 Immunohistochemistry (IHC) and hematoxylin-eosin (H&E) staining

The tumors were fixed in 4 % paraformaldehyde overnight, followed by paraffin embedding. Sections (8 μm) were deparaffinized in xylene and rehydrated through a graded series of alcohols. Endogenous peroxidase activity was blocked by treatment with 3 % hydrogen peroxide for 5 min, and all slides were subjected to heat-induced antigen retrieval by boiling in 10 mM citrate buffer (pH 6.0) for 10 min. Specific primary antibodies were applied and the slides were subsequently counterstained with hematoxylin. For H&E staining, the sections were immersed in hematoxylin for 3 min, rinsed with water for 30 min, and then stained with eosin for 3 min. Following dehydration in graded ethanol solutions, coverslips were mounted on the slides and examined using an optical microscope.

### 2.18 Statistical analysis

Unpaired two-tailed Student's t-test and one- and two-way analysis of variance (ANOVA) were used to calculate P values by GraphPad Prism 8. The Tukey method was used to adjust multiple comparisons. Quantifications were performed from at least three independent experiments. All data represent by mean ± SD except where specially indicated as mean ± SEM in the figure legends. P < 0.05 was considered significant.

## 3 Results

### 3.1 SFN induces ferroptosis in OS cells

To fully achieve the anti-tumor efficacy of SFN in human OS, we firstly examine the cytotoxicity of SFN in 143B and SJSA-1 human OS cells. Data showed that SFN inhibited the proliferation of 143B and SJSA-1 cells in dose- and time-dependent manner (Fig. 1B). Furthermore, colony formation assay demonstrated a significant reduction in the proliferative capacity of both 143B and SJSA-1 cells (Fig. 1C).

To identify the primary cell death type triggered by SFN, specific inhibitors of necroptosis, autophagy, apoptosis, and ferroptosis were employed. With the co-treatment of SFN/inhibitor, data showed that Nec-1 (necrostatin-1, necroptosis inhibitor), Z-VAD (Z-VAD-FMK, pan-caspase inhibitor), and CQ (chloroquine, autophagy inhibitor) could not prevent SFN-induced cell death in 143B and SJSA-1 cells (Figs. S1A–C). However, the ferroptosis inhibitors Fer-1 (ferrostatin-1), DFO (deferoxamine), and Lip-1 (liproxstatin-1) substantially rescued the cells from SFN-induced cell death (Fig. 1D, Figs. S1D and E), which indicated that ferroptosis was the predominant cell death form of OS cells treated by SFN. To further confirm the induction of ferroptosis in OS cells in response to SFN, we assessed key events associated with ferroptosis process, including ROS accumulation, GSH depletion, and lipid peroxidation. Data showed that SFN treatment led to elevated levels of ROS and increased lipid peroxidation (Fig. 1E and F), along with a significant reduction in GSH levels (Fig. 1G). Additionally, transmission electron microscopy (TEM) revealed morphological changes consistent with ferroptosis in SFN-treated cells, including altered mitochondrial structure, increased mitochondrial membrane density, and reduced or absent mitochondrial cristae (Fig. 1H). Moreover, western blotting analysis demonstrated that SFN treatment markedly reduced the expression levels of ferroptosis markers GPX4 and SLC7A11 in OS cells (Fig. 1I). These data indicate that SFN exerts potent pro-ferroptotic effect in OS cells.

### 3.2 SFN-mediated cytotoxicity in OS cells is dependent on ferroptosis

**Fig. 2:**
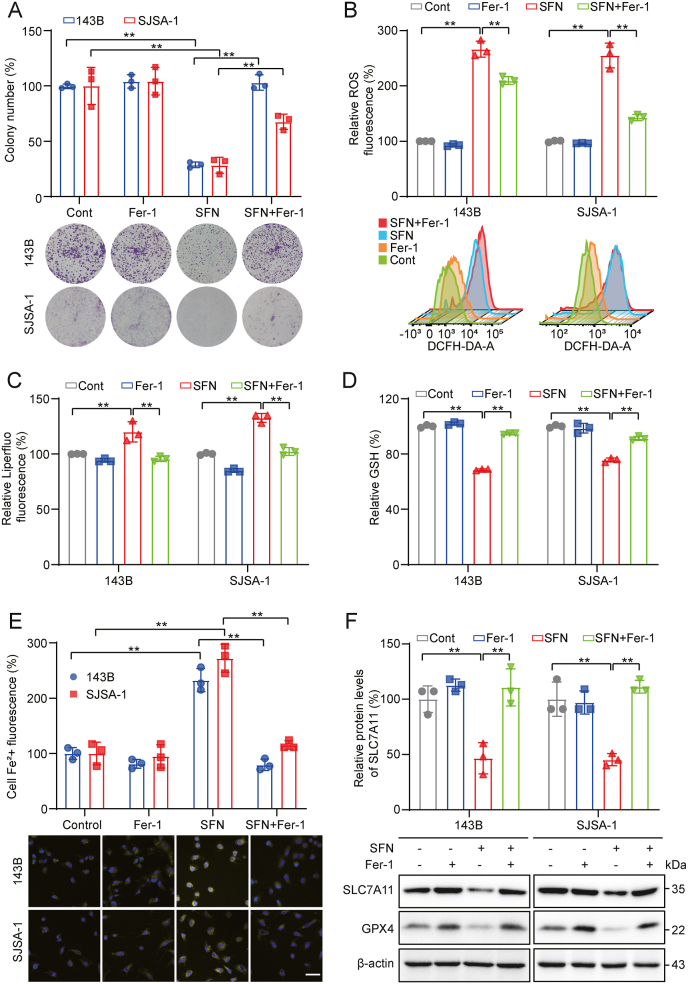
**Fer-1 reverses SFN-induced ferroptosis in OS cells. (A)** Treatment of 143B and SJSA-1 cells with SFN (1 μM) with or without Fer-1 (1 μM), with representative results of colony formation and quantitative analysis. **(B)** Analysis of ROS levels in 143B and SJSA-1 cells treated with SFN (10 μM) with or without Fer-1 (5 μM). **(C)** Analysis of Liperfluo levels in 143B and SJSA-1 cells treated with SFN (10 μM) with or without Fer-1 (5 μM). **(D)** Detection of GSH levels in 143B and SJSA-1 cells treated with SFN (10 μM) with or without Fer-1 (5 μM). **(E)** Detection of intracellular Fe^2+^ levels in 143B and SJSA-1 cells treated with SFN (10 μM) with or without Fer-1 (5 μM). Scale bar: 200 μm. **(F)** Western blotting to detect the expression of ferroptosis-related proteins in OS cells after 12 h treatment with SFN (10 μM) with or without Fer-1 (5 μM). ∗*P* < 0.05, ∗∗*P* < 0.01.

To determine the role of ferroptosis in SFN-mediated inhibition of OS, a series of rescue experiments were conducted using the ferroptosis inhibitor Fer-1. Colony formation assay showed that with the treatment of Fer-1, SFN induced suppression of 143B and SJSA-1 cells was significantly abolished (Fig. 2A). Additionally, Fer-1 alleviated SFN-induced ROS accumulation, lipid peroxidation, and GSH depletion (Fig. 2B–D). Furthermore, FerroOrange fluorescent probe validated the intracellular Fe^2+^ accumulation by SFN, as well as the abolishment of the increased Fe^2+^ levels by Fer-1 (Fig. 2E). Moreover, Fer‐1 rescued the reduction of SLC7A11 and GPX4 induced by SFN (Fig. 2F). These findings demonstrate that SFN effectively induces ferroptosis, acting as the primary anti-tumor effect in OS.

### 3.3 SFN induces ferroptosis of OS cells through downregulation of SLC7A11

**Fig. 3:**
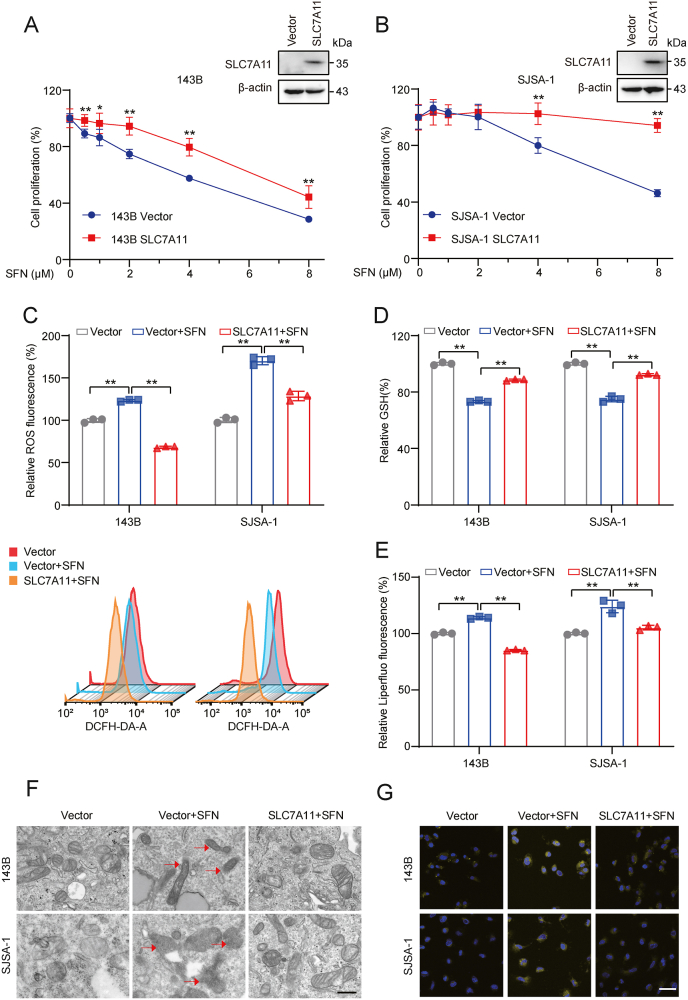
**SLC7A11 is accounted for SFN-induced ferroptosis in OS cells. (A, B)** Treatment of 143B (A) and SJSA-1 (B) cells with SFN for 48 h with or without overexpression of SLC7A11, followed by cell viability assay. **(C)** Treatment of 143B and SJSA-1 cells with SFN (10 μM) for 12 h with or without overexpression of SLC7A11, and subsequent assay of relative ROS levels. **(D)** Treatment of 143B and SJSA-1 cells with SFN (10 μM) for 12 h with or without overexpression of SLC7A11, followed by assay of relative GSH levels. **(E)** Treatment of 143B and SJSA-1 cells with SFN (10 μM) for 12 h with or without overexpression of SLC7A11, and subsequent assay of relative Liperfluo levels. **(F)** Treatment of 143B and SJSA-1 cells with SFN (10 μM) for 12 h with or without overexpression of SLC7A11, and image of transmission electronic microscopy. Red arrow: mitochondrial change in SFN-treated cells. Scale bar: 500 nm. **(G)** Treatment of 143B and SJSA-1 cells with SFN (10 μM) for 12 h with or without overexpression of SLC7A11, and subsequent assay of intracellular Fe^2+^ levels. Scale bar: 200 μm ∗*P* < 0.05, ∗∗*P* < 0.01.

Lipid peroxidation, the initiating step of ferroptosis, is characterized by decreased GSH which is a crucial antioxidant that helps role in maintaining the redox balance and defending against oxidative stress in cells [42]. SLC7A11, a key component of the cystine-glutamate antiporter regulating GSH generation, was downregulated notably in SFN-treated OS cells, which was more prominent than GPX4 (Fig. 1I). Since SLC7A11 is the upstream regulator of GPX4, we further elucidate the role of SLC7A11 in the anti-cancer effect of SFN against OS. While overexpression of SLC7A11 did not affect cell proliferation (Fig. S2A), it significantly attenuated SFN-induced inhibition of proliferation in OS cells (Fig. 3A and B). Furthermore, SLC7A11 overexpression notably reversed SFN-induced changes in the ROS level, GSH level, and lipid peroxidation (Fig. 3C–E). Additionally, TEM and Fe^2+^ FerroOrange fluorescent analysis showed that the SFN-induced morphological changes of ferroptosis and enhanced Fe^2+^ levels could be abolished by overexpression of SLC7A11 (Fig. 3F and G). These results demonstrate the dependency of downregulation of SLC7A11 in SFN-induced ferroptosis in OS cells.

### 3.4 SFN downregulates SLC7A11 through induction of its autolysosomal degradation in OS cells

**Fig. 4:**
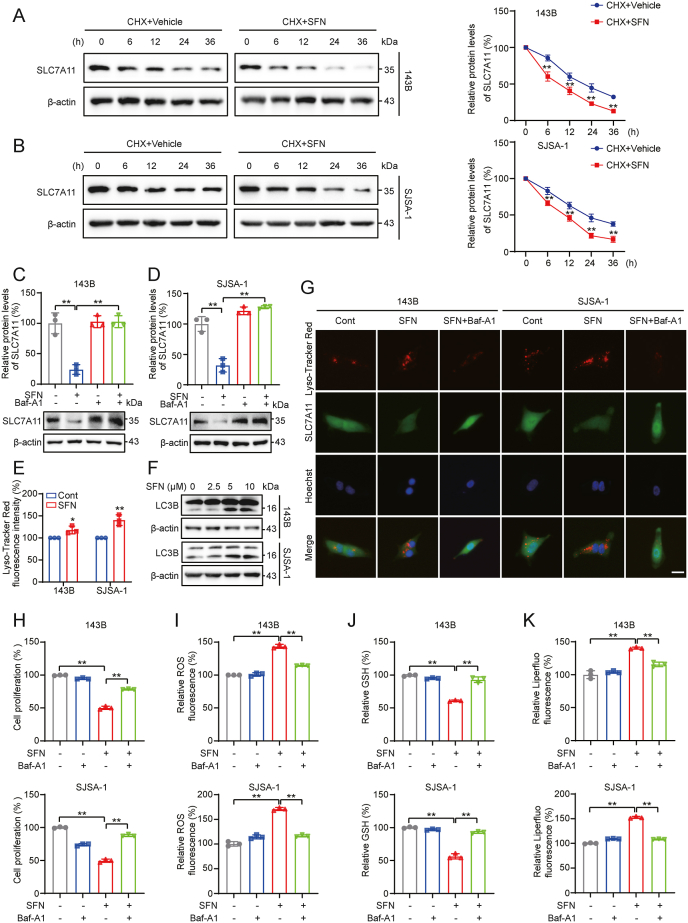
**SFN downregulates SLC7A11 via autophagy-lysosome degradation in OS cells. (A, B)** 143B (A) and SJSA-1 (B) cells treated with CHX (50 μg/ml) with or without SFN (5 μM), and collected at specified times. The cells were lysed, and the cell lysates were subsequently blotted with the indicated antibodies. **(C, D)** SLC7A11 expressions with SFN (10 μM) and Baf-A1 (200 nM) treatment in 143B (C) and SJSA-1 (D) cells. **(E)** 143B and SJSA-1 cells treated with SFN (10 μM) for 12 h. The cells were then loaded with LysoTracker Red reagent for 15 min. Fluorescence intensity was measured by flow cytometry. **(F)** Western blotting analysis of LC3B in 143B and SJSA-1 cells after treatment with indicated doses of SFN. **(G)** Representative image of the colocalization of SLC7A11 with lysosome in 143B and SJSA-1 cells. Scale bar: 20 μm. **(H)** 143B and SJSA-1 cells were treated with SFN (5 μM or 10 μM) with or without Baf-A1 (200 nM), and cell proliferation was analyzed. **(I)** 143B and SJSA-1 cells were treated with SFN (10 μM) with or without Baf-A1 (200 nM), and the ROS levels were analyzed. **(J)** 143B and SJSA-1 cells were treated with SFN (10 μM) with or without Baf-A1 (200 nM), and the GSH levels were detected. **(K)** 143B and SJSA-1 cells were treated with SFN (10 μM) with or without Baf-A1 (200 nM), and the Liperfluo levels were analyzed. ∗*P* < 0.05, ∗∗*P* < 0.01.

To explore the mechanism by which SFN decreases SLC7A11 expression, we first evaluated SLC7A11 mRNA levels in OS cells following SFN treatment. The results showed that SFN did not significantly reduce SLC7A11 mRNA levels in OS cells (Fig. S2B), indicating that the reduction in SLC7A11 expression is independent of transcriptional inhibition. Subsequently, we investigated whether the SFN-induced downregulation of SLC7A11 was due to enhanced protein degradation by measuring the half-life of the SLC7A11 protein using cycloheximide (CHX) chase assay. Results showed that the half-life of endogenous SLC7A11 protein was significantly reduced in OS cells subjected to SFN treatment (Fig. 4A and B). Furthermore, to identify the degradation mechanism of SLC7A11, we treated cells with SFN in combination with either bafilomycin A1 (Baf-A1), a lysosomal fusion inhibitor, or MG132, a proteasomal inhibitor. Western blotting analysis revealed that MG132 did not block SFN-induced degradation of SLC7A11 (Figs. S3A and B), whereas Baf-A1 completely inhibited it (Fig. 4C and D), suggesting that SLC7A11 is degraded predominantly by lysosomal degradation. Furthermore, to assess the effect of SFN on lysosomal activation, we performed LysoTracker assays. The fluorescence intensity of the pH-sensitive dye LysoTracker Red increased significantly with SFN treatment, indicating enhanced lysosomal acidification (reduced pH) (Fig. 4E). Additionally, SFN treatment significantly increased the autophagosome marker LC3II (Fig. 4F). Co-localization between SLC7A11 and the LysoTracker Red further supported enhanced interaction following SFN treatment, which could be inhibited by Baf-A1 treatment (Fig. 4G). Moreover, to further confirm the functional role of autophagy-lysosomal pathway in the activity of SFN against OS, a series of rescue experiments using Baf-A1 was performed. Results showed that Baf-A1 demonstrated restoration of cell proliferation in 143B and SJSA-1 cells treated with SFN (Fig. 4H). Baf-A1 also partially reversed SFN-induced ROS accumulation, GSH depletion, and lipid peroxidation in OS cells (Fig. 4I–K). Collectively, these findings suggest that SFN promotes SLC7A11 degradation through the autophagy-lysosome pathway.

### 3.5 SFN downregulates SLC7A11 via p62-dependent selective autophagy

**Fig. 5:**
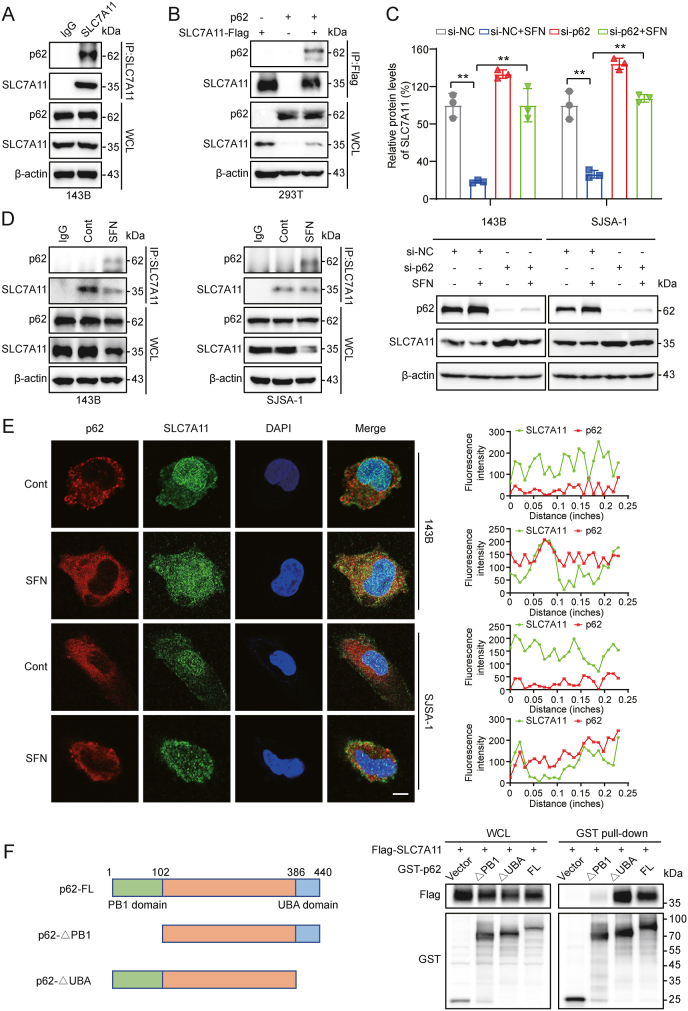
**SFN regulates SLC7A11 via p62 dependent selective autophagy. (A)** Endogenous p62 was immunoprecipitated with SLC7A11 antibody and examined by immunoblotting. **(B)** Interaction between exogenous p62 and SLC7A11. HEK293T cells were co-transfected with indicated constructs. Cellular extracts were immunoprecipitated with FLAG Sepharose and immunoprecipitations were performed with antibodies against the indicated proteins. **(C)** Western blotting data from p62 siRNA assay, untreated or treated with SFN (10 μM) for 12 h. **(D)** Analysis of the interaction between SLC7A11 and p62. Cells left untreated or treated with SFN (10 μM) for 12 h. Equal amounts of cell lysates were subjected to immunoprecipitation (IP) with antibodies against SLC7A11 or control IgG and western blotted with the respective antibodies. **(E)** Representative image and colocalization efficiency were measured by ImageJ software of immunofluorescence staining in 143B and SJSA-1 cells for immunostaining of SLC7A11 (green) with p62 (red). The fluorescence-integrated density was measured by ImageJ software. Scale bar: 5 μm. **(F)** Identification of PB1 domain of p62 is responsible for the interaction with SLC7A11. Flag-SLC7A11 construct was cotransfected with various truncates of GST tagged p62 into HEK293T cells. Cell lysates were pulled down with glutathione beads and analyzed by western blotting. ∗*P* < 0.05, ∗∗*P* < 0.01.

It has been reported that selective autophagic degradation is closely related ferroptosis [43]. We further examined the potential role of some major autolysosomal proteins including autophagy-related protein 3 (ATG3), lysosome associated membrane protein 1 (LAMP1), LC3B, and p62 in selective autophagy related SLC7A11 degradation. Data from Co-IP assay showed that among these autophagy-related proteins, the adapter protein p62 selectively interacted with SLC7A11 in OS cells (Fig. 5A; Fig. S4), which was confirmed by pull-down assay with exogenous expressed proteins (Fig. 5B). Subsequently, we further determine the role of p62 in SFN-induced downregulation of SLC7A11. As shown in Fig. 5C, silencing p62 reversed SFN-induced decrease in SLC7A11 protein levels (Fig. 5C). Additionally, CHX assay also demonstrated that the degradation of SLC7A11 induced by SFN in OS cells was reversed by si-p62 treatment (Fig. S5). Immunoprecipitation assay revealed that SFN treatment increased the binding of p62 to SLC7A11 in both 143B and SJSA-1 cells (Fig. 5D). Immunofluorescence co-staining also showed a significant increase in the colocalization of SLC7A11 with p62 following SFN treatment (Fig. 5E). To identify the binding domain of p62 responsible for its interaction with SLC7A11, we conducted truncation GST pull-down assay with p62 truncation variants [44]. The results indicated that the PB1 domain of p62 is responsible for the interaction with SLC7A11 (Fig. 5F). These results indicate that p62 plays a crucial role in the degradation of SLC7A11.

### 3.6 SFN directly binds to autophagic cargo adapter p62 in OS cells

**Fig. 6:**
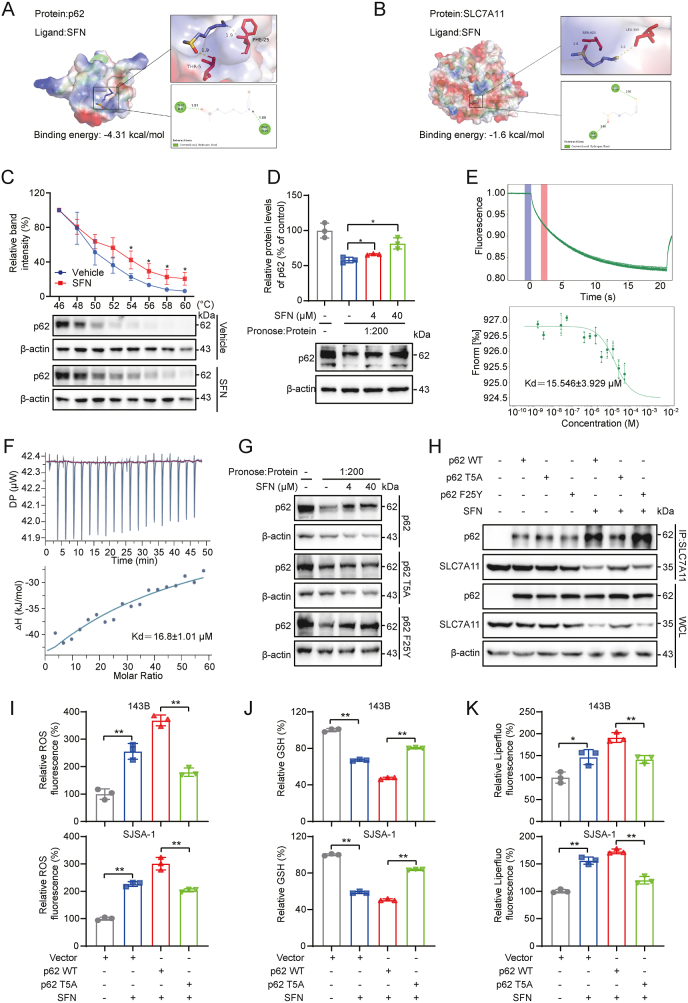
**SFN binds to the p62 protein. (A)** Predicted binding mode of SFN with p62. **(B)** Predicted binding mode of SFN with SLC7A11. **(C)** OS cells were treated with PBS or SFN (20 μM) for 2 h and then heated at a gradient of 46∼60 °C. Soluble p62 was detected by western blotting. **(D)** The binding of SFN to p62 was determined by DARTS approach. **(E)** The binding affinity between SFN (500,00–1.53 nM) and p62 was determined using MST. **(F)** The thermogram and binding isotherm for the titration of SFN with p62. **(G)** The interactions of SFN with p62 mutants in OS cells were determined by DARTS approach. **(H)** OS cells were transfected with wild-type or mutant p62 for 24 h and were further treated with or without SFN. Then, the interaction between p62 and SLC7A11 was determined by western blotting. **(I–K)** 143B and SJSA-1 cells were transfected with either p62-WT or p62 T5A mutant, followed by treatment with SFN (10 μM). ROS levels (I), GSH levels (J), and Liperfluo levels (K) were analyzed, respectively. ∗*P* < 0.05, ∗∗*P* < 0.01.

To elucidate the specific mechanism by which SFN regulates p62 and SLC7A11, we hypothesize that SFN might directly bind either p62 or SLC7A11, conferring the association between p62 and SLC7A11. To test this, we first conducted a molecular docking to achieve a binding profile of SFN and proteins. Data showed that a direct binding between SFN and p62 with the binding energy of −4.31 kcal/mol, which is smaller than that of SFN and SLC7A11 (Fig. 6A and B). Subsequently, we investigated the thermal stabilization of p62 upon SFN binding using the Cellular Thermal Shift Assay (CETSA) and found SFN significantly enhanced the thermal stability of p62 in OS cells (Fig. 6C), while it had no effect on the stability of SLC7A11 (Fig. S6). Furthermore, Drug Affinity Responsive Target Stability (DARTS) assay confirmed the binding of SFN to p62 (Fig. 6D). For the quantified analysis of the binding of SFN and p62, Microscale Thermophoresis (MST) analysis showed a dose-dependent interaction of SFN and p62, with a dissociation constant (Kd) of 15.546 ± 3.929 μМ (Fig. 6E) which was similar with that from Isothermal Titration Calorimetry (ITC) analysis (Fig. 6F). These data establish that SFN directly binds to p62.

Furthermore, to validate the SFN-p62 interaction as well as its role in regulation of SLC7A11, mutation verification tests were conducted based the predicted binding sites of SFN at p62 Thr5 (T5) and Phe25 (F25) residues on p62 (Fig. 6A). With 143B cells transfected with wild-type p62 (WT), or p62 with T5A mutation (T5A), or p62 with F25Y mutation (F25Y), DARTS analysis showed that compared to WT and F25Y, T5A abolished the binding to SFN which did not protect this mutant from pronase-mediated degradation (Fig. 6G) in OS cells. IP analysis showed that SFN significantly increased the interaction between p62 and SLC7A11 in cells expressing WT or the F25Y mutant, but not in cells expressing the T5A mutant (Fig. 6H). Moreover, compared to WT and F25Y, the T5A mutant significantly reversed the downregulation of SLC7A11 in OS cells (Fig. 6H). Further measurement of ROS levels, GSH levels, and lipid peroxidation in cells demonstrated that the ferroptosis-inducing effects by SFN were abolished by T5A mutant (Fig. 6I–K). These findings indicate that SFN binding to Thr5 of p62 promotes the interaction between p62 and SLC7A11, leading to the degradation of SLC7A11, thereby inducing ferroptosis.

### 3.7 SFN blocks OS progression through induction of ferroptosis *in vivo*

**Fig. 7:**
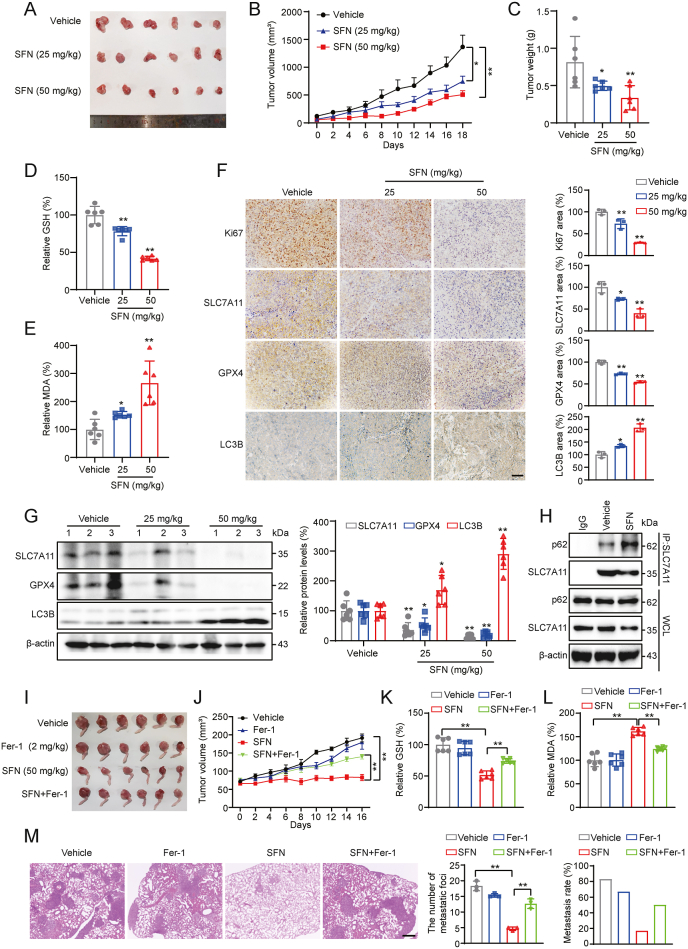
**SFN blocks OS progression through induction of ferroptosis. (A)** Representative photograph of tumor samples from each group. **(B)** Measurement of tumor volume. Data are presented as mean ± SEM. **(C)** Measurement of tumor weight. **(D)** Analysis of GSH levels in tumor tissues. **(E)** Analysis of MDA levels in tumor tissues. **(F)** IHC to determine the levels of Ki67, SLC7A11, GPX4, and LC3B in tumor tissues. Scale bar: 200 μm. **(G)** Measurement of protein expression levels of SLC7A11, GPX4, and LC3B in tumor tissues. **(H)** SFN induces the interaction of SLC7A11 and p62 *in vivo* assayed by Co-IP assay. **(I)** Representative photograph of tumor samples from each group. **(J)** Measurement of tumor volumes. Data are presented as mean ± SEM. **(K)** Analysis of GSH levels in tumor tissues. **(L)** Analysis of MDA levels in tumor tissues. **(M)** Lung tissues were harvested and subjected to H&E staining. Quantification of the number of lung metastatic foci and lung metastasis rate in each group is shown. Scale bar: 500 μm ∗*P* < 0.05, ∗∗*P* < 0.01.

To assess the antitumor effects of SFN as well as the role of ferroptosis *in vivo*, two OS xenograft models were established. In the subcutaneous tumor model, following SFN treatment, the tumor volume and tumor weight were suppressed dose-dependently (Fig. 7A–C). SFN treatment reduced GSH levels and increased MDA production, indicating the induction of ferroptosis (Fig. 7D and E). Additionally, immunohistochemistry (IHC) and western blot analyses of subcutaneous tumors revealed significantly lower expression levels of Ki67, SLC7A11, and GPX4, along with upregulated LC3B in the SFN-treated group (Fig. 7F and G). Co-IP experiment confirmed that SFN promoted the p62/SLC7A11 interaction in tumors (Fig. 7H). These data suggest that SFN exerts an antitumor effect in OS *in vivo*, which is associated with the induction of ferroptosis.

To determine the dependency of ferroptosis in SFN-induced blockade of OS progression, an intratibial xenograft mouse model and the ferroptosis inhibitor Fer-1 were employed. Following treatment with SFN or/and Fer-1, the data showed that SFN significantly inhibited OS growth and Fer-1 notably attenuated the SFN-induced inhibition in the orthotopic xenograft models (Fig. 7I and J). Consistent with the subcutaneous tumor, the GSH levels and MDA production were decreased and increased, respectively (Fig. 7K and L). Similar with the data from subcutaneous tumor model, IHC and western blotting analyses revealed significantly lower expression levels of Ki67, SLC7A11, and GPX4 in the SFN treatment group (Figs. S7A and B). Moreover, compared to the vehicle group, SFN treatment significantly reduced the number of lung metastatic foci, and Fer-1 partially reversed blockade of metastasis by SFN (Fig. 7M). Moreover, for the two *in vivo* models, no significant difference in body weight was observed between the control and SFN-treated groups (Figs. S7C and D), and pathological examination of major organs (liver, kidney, heart, spleen, and lung) showed no evidence of toxicity associated with SFN treatment (Fig. S7E). Collectively, these findings indicate that SFN blocks OS progression associated with its induction of ferroptosis *in vivo*.

## 4 Discussion

OS is the most prevalent bone tumor in children and adolescents [45], with a bimodal age distribution. Despite the use of chemotherapy (cisplatin, doxorubicin, and methotrexate), surgery, and radiation therapy, survival rates have not improved over the past four decades [46]. Therefore, the search for safe and effective drugs, along with the identification of novel therapeutic targets for OS is of paramount importance.

OS treatment faces several unique challenges, including high metastatic potential, especially to the lungs, and the development of drug resistance to conventional chemotherapy [47,48]. Patients often experience severe side effects due to the high toxicity of current treatments. Therefore, treatment drugs must effectively inhibit tumor growth and metastasis, overcome drug resistance, minimize toxicity, and be compatible with combination therapies to enhance outcomes. Currently, natural plant extracts are considered an important source of anti-cancer agents due to their potential biological activity, reduced toxicity, and side effects compared to traditional chemotherapeutic drugs. In the present study, we report that SFN, an isothiocyanate derived from cruciferous vegetables, exhibits potent anti-tumor activities in OS cells by inducing ferroptosis both *in vitro* and *in vivo*. Mechanistically, SFN directly binds with p62, enhancing the interaction between p62 and SLC7A11, leading to SLC7A11 degradation through the autophagic lysosomal pathway.

Ferroptosis, a recently discovered programmed cell death form, has emerged as a key focus in cancer research. It is primarily driven by lipid metabolism, GSH depletion, and dysregulated iron metabolism [49]. The induction of ferroptosis has been recognized as a promising therapeutic strategy for cancer treatment. SFN offers several advantages in cancer treatment by inducing ferroptosis. Based on our findings, its ferroptosis-inducing activity indicates it selectively targets cancer cells that rely on iron metabolism and ROS generation, with minimal impact on normal cells, reducing harm to healthy tissues. Unlike traditional chemotherapy, its multi-target mechanism lowers the risk of drug resistance, especially in chemotherapy-resistant tumors. As a natural compound, SFN is well-tolerated with fewer side effects and toxicity [50], which has been confirmed in our *in vivo* experiments. Additionally, it exhibits multiple anti-cancer mechanisms, such as antioxidant, anti-inflammatory, and cell cycle regulation, enhancing its therapeutic potential [[51], [52], [53]]. SLC7A11, a key regulator of ferroptosis that is often overexpressed in many cancers, plays a crucial role in promoting GSH biosynthesis and ferroptosis resistance [54]. While its expression is regulated by multiple mechanisms, including transcriptional regulation by transcription factors [55] epigenetic regulators [56], and post-transcriptional regulatory mechanisms that modulate protein stability [57,58], subcellular localization [59], and transporter activity [60]. Our findings show that SFN downregulates SLC7A11 protein levels without affecting its mRNA transcription. This led us to investigate SFN's role in SLC7A11 protein degradation, which we found to occur through the lysosomal pathway, as the lysosomal inhibitor Baf-A1, but not the proteasome inhibitor MG132, blocked its degradation. Although the autophagic degradation of SLC7A11 has been reported [61], our further studies revealed that the adapter protein p62 interacted with SLC7A11 and conferred its autophagic degradation. Moreover, SFN enhances the binding between p62 and SLC7A11, facilitating the selective autophagic degradation of SLC7A11. Importantly, we demonstrated that SFN directly binds to p62, driving the degradation of SLC7A11 through the lysosomal pathway.

In two *in vivo* OS xenograft models, SFN's antitumor efficacy was confirmed, with the induction of ferroptosis observed, and the ferroptosis inhibitor Fer-1 partially rescuing this effect. These findings collectively underscore SFN's potential as a ferroptosis inducer and a promising therapeutic agent for OS treatment. For the treatment of OS in clinic, particularly for the combination treatment, SFN can enhance the therapeutic effect by synergizing with chemotherapy or immunotherapy, potentially overcoming drug resistance by targeting key ferroptosis-related molecules like SLC7A11. As a natural compound, SFN has lower toxicity compared to traditional chemotherapeutics, allowing for reduced doses of standard drugs and minimizing side effects. Additionally, SFN acts through different oncogenic pathways, complementing other treatments, and it can inhibit both tumor growth and metastasis, reducing the risk of recurrence. Thus, SFN holds promise as a valuable adjunct in OS therapy.

## 5 Conclusion

**Fig. 8:**
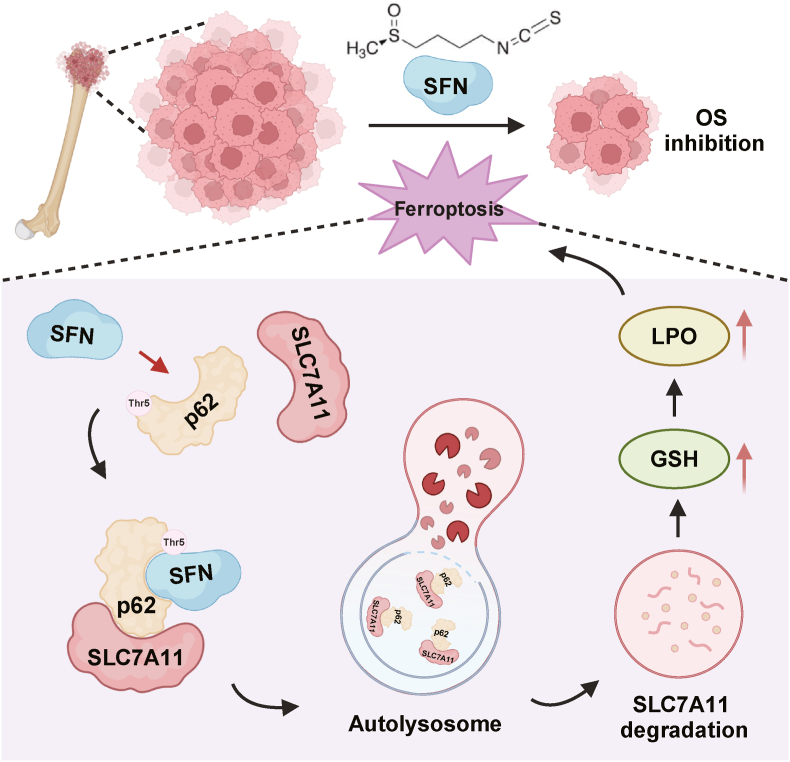
**Schematic illustration of SFN-induced ferroptosis through downregulation of SLC7A11 by interacting with p62 in OS cells.** In OS cells, SFN directly interacts with p62, promotes the association of p62 and SLC7A11, leading to autolysosomal degradation of SLC7A11, subsequently promoting ROS production, lipid peroxidation, ultimately inducing ferroptosis.

In conclusion, we reveal that p62 play an important role for SLC7A11 autophagic degradation and our data suggest that SFN exerts its anti-osteosarcoma effects by inducing ferroptosis through targeting p62, leading to the degradation of SLC7A11 via the lysosomal pathway (Fig. 8). These findings provide new evidence for targeting SLC7A11 to treat cancer and offer the foundation for application of SFN as therapeutic agent for the treatment of OS.

## CRediT authorship contribution statement

**Qiuming Zou:** Writing – review & editing, Writing – original draft, Visualization, Validation, Methodology, Investigation, Formal analysis. **Xiaofeng Zhou:** Writing – review & editing, Writing – original draft, Validation, Methodology, Investigation, Formal analysis. **Jianqin Lai:** Validation, Resources, Methodology, Investigation, Data curation. **Haixia Zhou:** Methodology, Investigation. **Jinxuan Su:** Writing – review & editing, Methodology, Investigation. **Zhijing Zhang:** Validation, Methodology, Investigation. **Xiaosong Zhuang:** Visualization, Methodology, Investigation. **Lili Liu:** Methodology, Investigation. **Ruijie Yuan:** Software, Project administration. **Sijia Li:** Validation, Resources. **Siyu Yang:** Validation, Software. **Xinyi Qu:** Validation, Resources. **Jiezhu Feng:** Methodology, Investigation. **Yongqi Liu:** Project administration, Data curation. **Zisheng Li:** Visualization. **Shiting Huang:** Visualization. **Zhi Shi:** Resources, Methodology. **Yu Yan:** Supervision, Project administration, Conceptualization. **Zhiming Zheng:** Supervision, Project administration, Conceptualization. **Wencai Ye:** Supervision, Resources, Funding acquisition. **Qi Qi:** Writing – review & editing, Supervision, Resources, Funding acquisition, Conceptualization.

## Data availability

Data will be made available on request.

## Declaration of competing interest

The authors declare that they have no conflict of interest.

## Figures and Tables

**Table:** 

CETSA	Cellular thermal shift assay
CHX	C**y**cloheximide
DARTS	Drug affinity responsive target stability
DCFH	DA Dichlorodihydrofluorescein diacetate
GPX4	Glutathione peroxidase 4
GSH	Glutathione
HE	Hematoxylin and eosin
IHC	Immunohistochemitry
ITC	Isothermal Titration Calorimetry
LPO	Lipid peroxidation
MST	Microscale thermophoresis
MTT	Thiazolyl blue tetrazolium bromide
OS	Osteosarcoma
p62	Sequestosome-1
ROS	Reactive oxygen species
SFN	Sulforaphane

## Appendix A Supplementary data

Supplementary figure 1. Effect of SFN alone or combined with different cell death inhibitors on the cell viability of OS cells. (A) Treatment of 143B and SJSA-1 cells with SFN with or without Nec-1 (10 μM) for 48 h, followed by cell viability detection. (B) Treatment of 143B and SJSA-1 cells with SFN with or without Z-VAD (10 μM) for 48 h, and subsequent cell viability detection. (C) Treatment of 143B and SJSA-1 cells with SFN with or without CQ (25 μM) for 48 h, and cell viability detection. (D) 143B and SJSA-1 cells were exposed to SFN with or without DFO (50 μM) for 48 h, and cell viability was assessed. (E) 143B and SJSA-1 cells were treated with SFN with or without Lip-1 (1 μM) for 48 h, and cell viability was evaluated. ∗P < 0.05, ∗∗P < 0.01.

Supplementary figure 2. Effect of SLC7A11 on the proliferation and effect of SFN on the mRNA levels of SLC7A11 in OS cells. (A) 143B and SJSA-1 cells transfected SLC7A11. Cell proliferation was examined with MTT assay. (B) Relative mRNA levels of SLC7A11 were measured after SFN treatment. ∗P < 0.05, ∗∗P < 0.01.

Supplementary figure 3. Effect of MG132 on SFN-induced degradation of SLC7A11. Following treatment with SFN and/or MG132, 143B (A) and SJSA-1 (B) cells were collected and analyzed by western blot analysis. ∗P < 0.05, ∗∗P < 0.01.

Supplementary figure 4. Examination of the interactions between SLC7A11 and major autolysosomal proteins. With 143B cells, endogenous LC3B, ATG3, and LAMP1 were immunoprecipitated with SLC7A11 antibody and examined by western blot analysis.

Supplementary figure 5. Effect of si-p62 on SFN-regulated SLC7A11 protein stability. (A, B) Following transfection with si-NC or si-p62, 143B (A) and SJSA-1 (B) cells were treated with CHX (50 μg/ml) and SFN (5 μM) for the indicated durations. The cells were then lysed and subjected to western blotting with the indicated antibodies.

Supplementary figure 6. Effect of SFN on the interaction with SLC7A11 assayed by CESTA. 143B cells treated with PBS or SFN (20 μM) for 2 h and then heated at a gradient of 46∼60 °C. Soluble SLC7A11 was detected by western blotting. ∗P < 0.05, ∗∗P < 0.01.

Supplementary figure 7. Effect of SFN on OS xenograft *in vivo*. (A) IHC to determine the levels of Ki67, SLC7A11, and GPX4 in tumor tissues from intratibial xenograft mouse model. Scale bar: 200 μm. (B) The protein expression levels of SLC7A11 and GPX4 in tumor tissues intratibial xenograft mouse model. (C, D) The body weights of tumor bearing mice in subcutaneous (C) and intratibial (D) xenograft models were measured every other day. (E) H&E staining of liver, kidney, heart, spleen, and lung from animals treated with vehicle control or 25 or 50 mg/kg of SFN. Scale bar: 100 μm ∗*P* < 0.05, ∗∗*P* < 0.01.
