# Supplementary figures and images for: Targeting p62 by sulforaphane promotes autolysosomal degradation of SLC7A11, inducing ferroptosis for osteosarcoma treatment
Source: Redox Biol. 2024 Dec 5;79:103460. doi: 10.1016/j.redox.2024.103460 (PMC11681892; doi:10.1016/j.redox.2024.103460)

**A**

143B

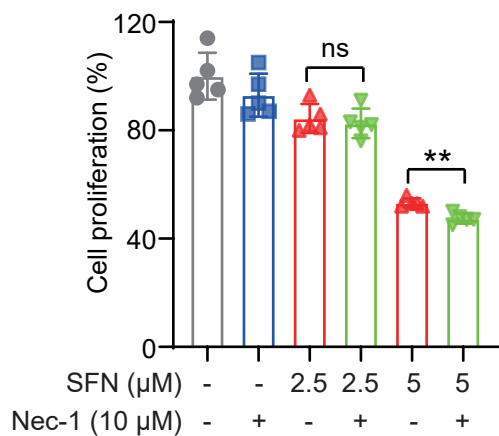**B**

143B

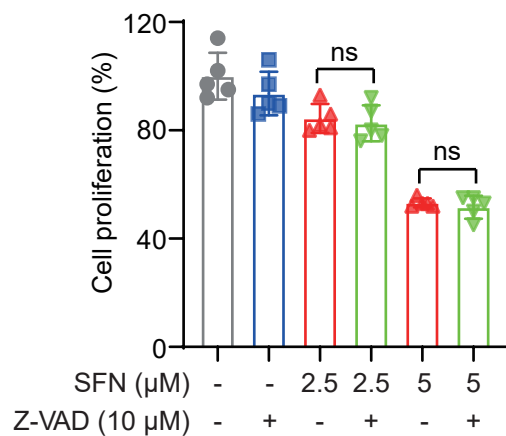**C**

143B

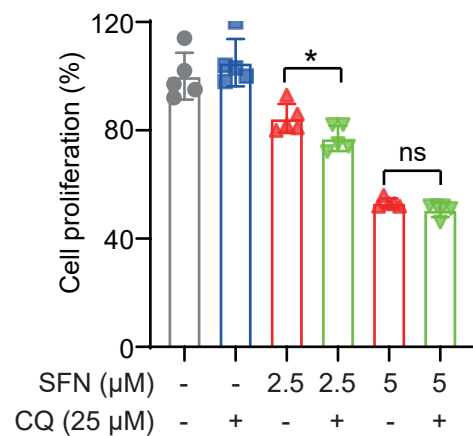

SJSA-1

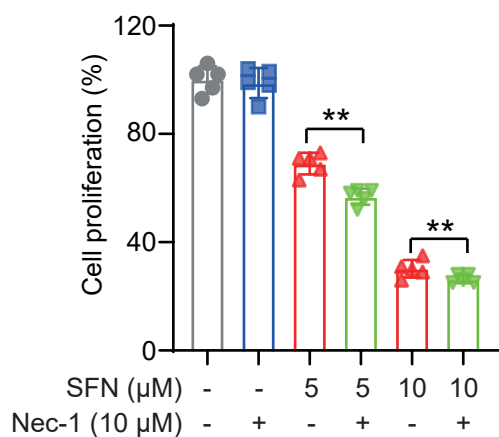

SJSA-1

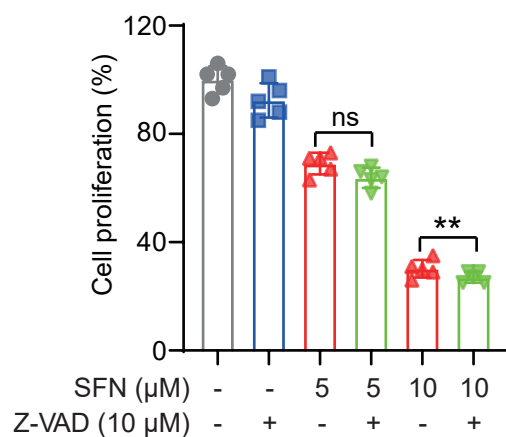

SJSA-1

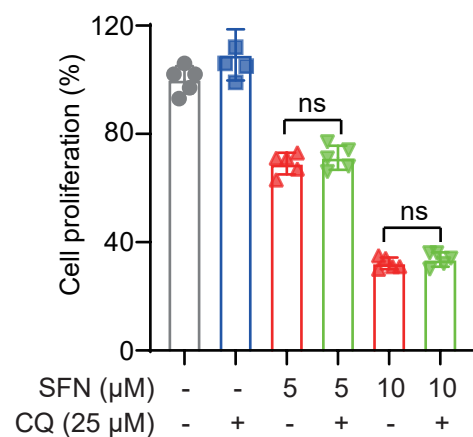**D**

143B

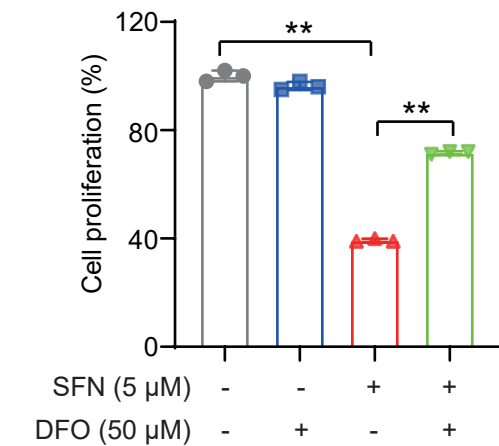**E**

143B

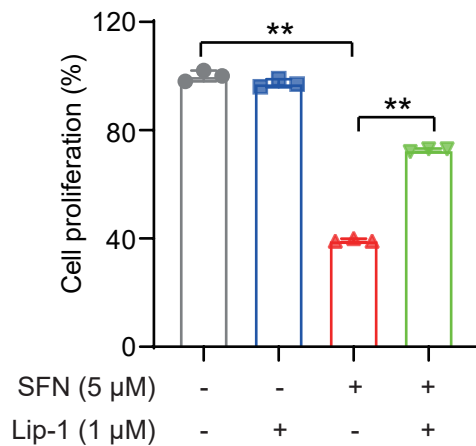

SJSA-1

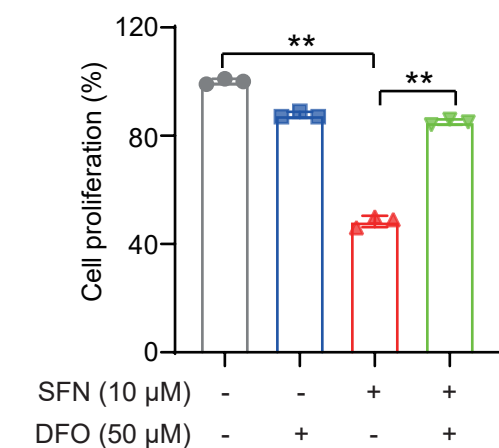

SJSA-1

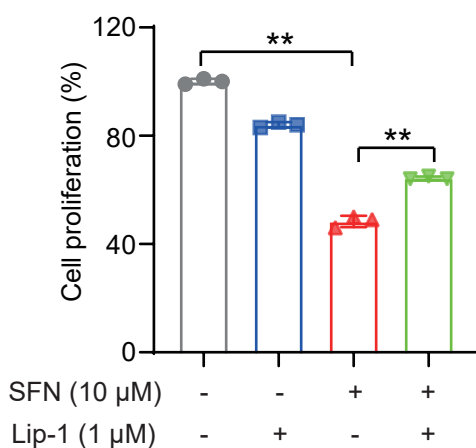

Supplement: Supplementary file 1 — Supplementary figure 1. Effect of SFN alone or combined with different cell death inhibitors on the cell viability of OS cells. (A) Treatment of 143B and SJSA-1 cells with SFN with or without Nec-1 (10 μM) for 48 h, followed by cell viability detection. (B) Treatment of 143B and SJSA-1 cells with SFN with or without Z-VAD (10 μM) for 48 h, and subsequent cell viability detection. (C) Treatment of 143B and SJSA-1 cells with SFN with or without CQ (25 μM) for 48 h, and cell viability detection. (D) 143B and SJSA-1 cells were exposed to SFN with or without DFO (50 μM) for 48 h, and cell viability was assessed. (E) 143B and SJSA-1 cells were treated with SFN with or without Lip-1 (1 μM) for 48 h, and cell viability was evaluated. ∗P < 0.05, ∗∗P < 0.01. [file mmc1.pdf]

**A**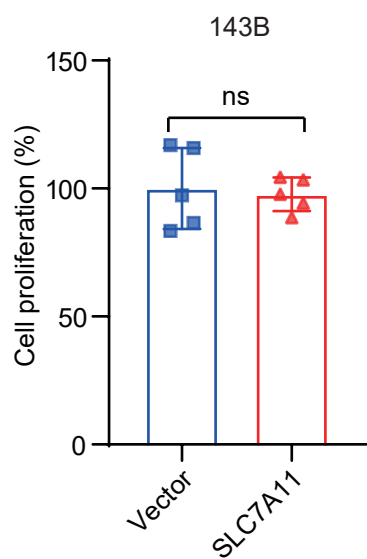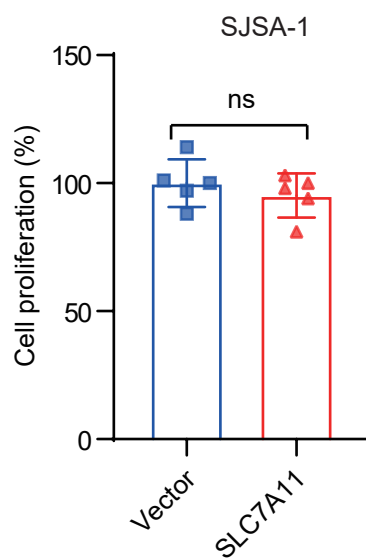**B**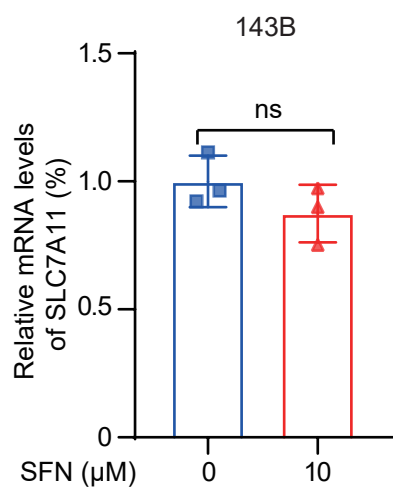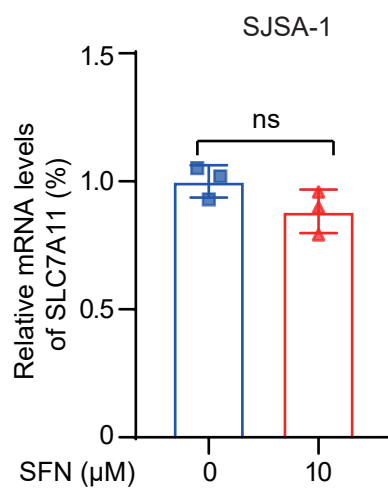

Supplement: Supplementary file 2 — Supplementary figure 2. Effect of SLC7A11 on the proliferation and effect of SFN on the mRNA levels of SLC7A11 in OS cells. (A) 143B and SJSA-1 cells transfected SLC7A11. Cell proliferation was examined with MTT assay. (B) Relative mRNA levels of SLC7A11 were measured after SFN treatment. ∗P < 0.05, ∗∗P < 0.01. [file mmc2.pdf]

**A**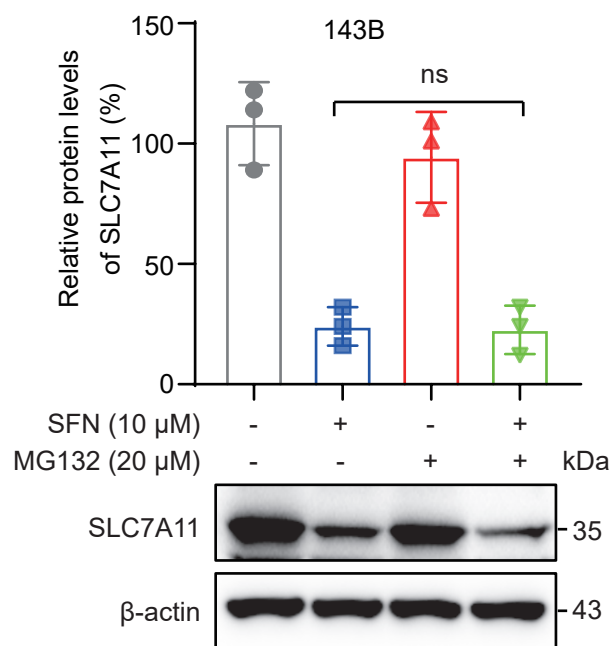**B**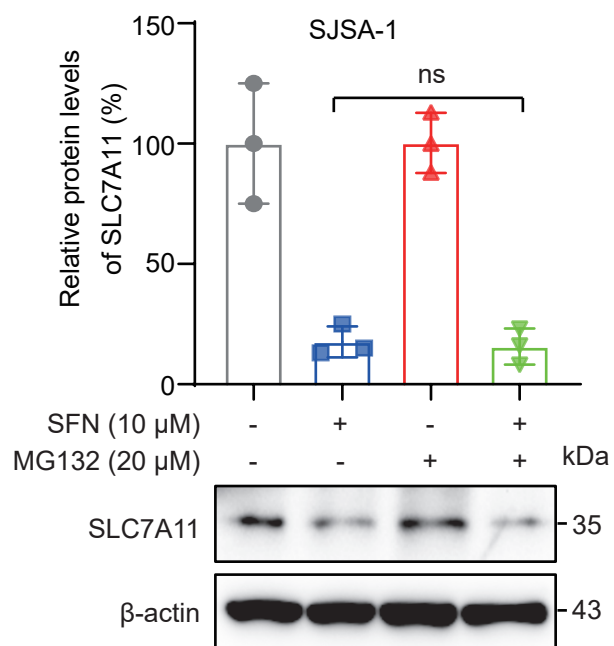

Supplement: Supplementary file 3 — Supplementary figure 3. Effect of MG132 on SFN-induced degradation of SLC7A11. Following treatment with SFN and/or MG132, 143B (A) and SJSA-1 (B) cells were collected and analyzed by western blot analysis. ∗P < 0.05, ∗∗P < 0.01. [file mmc3.pdf]

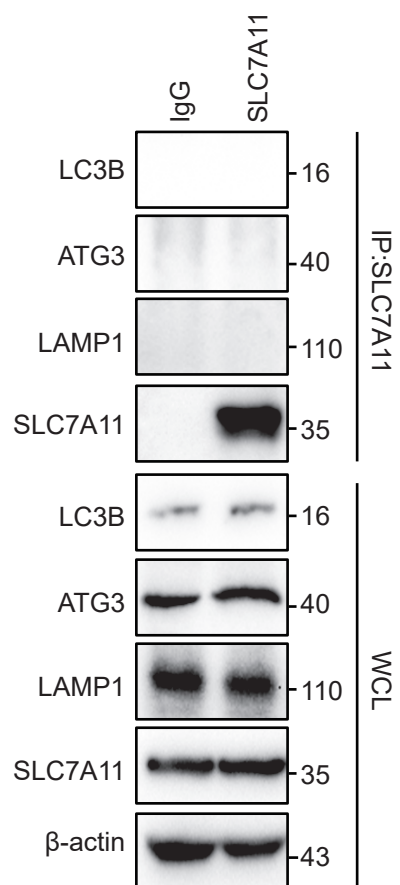

Supplement: Supplementary file 4 — Supplementary figure 4. Examination of the interactions between SLC7A11 and major autolysosomal proteins. With 143B cells, endogenous LC3B, ATG3, and LAMP1 were immunoprecipitated with SLC7A11 antibody and examined by western blot analysis. [file mmc4.pdf]

A

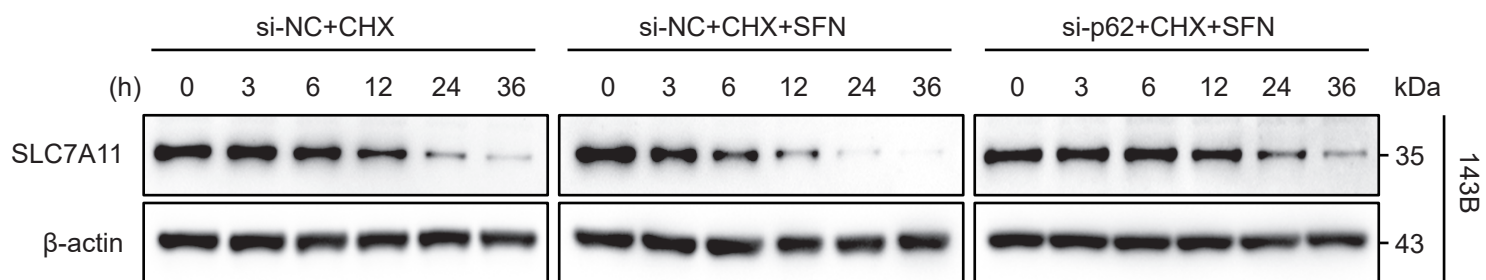

B

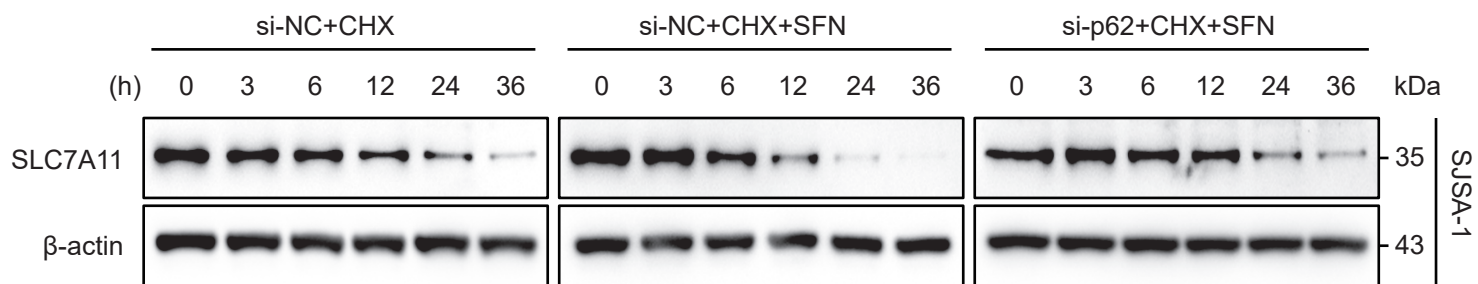

Supplement: Supplementary file 5 — Supplementary figure 5. Effect of si-p62 on SFN-regulated SLC7A11 protein stability. (A, B) Following transfection with si-NC or si-p62, 143B (A) and SJSA-1 (B) cells were treated with CHX (50 μg/ml) and SFN (5 μM) for the indicated durations. The cells were then lysed and subjected to western blotting with the indicated antibodies. [file mmc5.pdf]

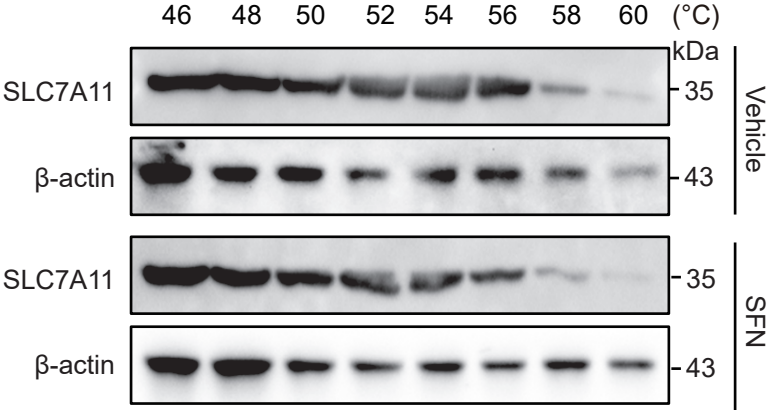

Supplement: Supplementary file 6 — Supplementary figure 6. Effect of SFN on the interaction with SLC7A11 assayed by CESTA. 143B cells treated with PBS or SFN (20 μM) for 2 h and then heated at a gradient of 46∼60 °C. Soluble SLC7A11 was detected by western blotting. ∗P < 0.05, ∗∗P < 0.01. [file mmc6.pdf]

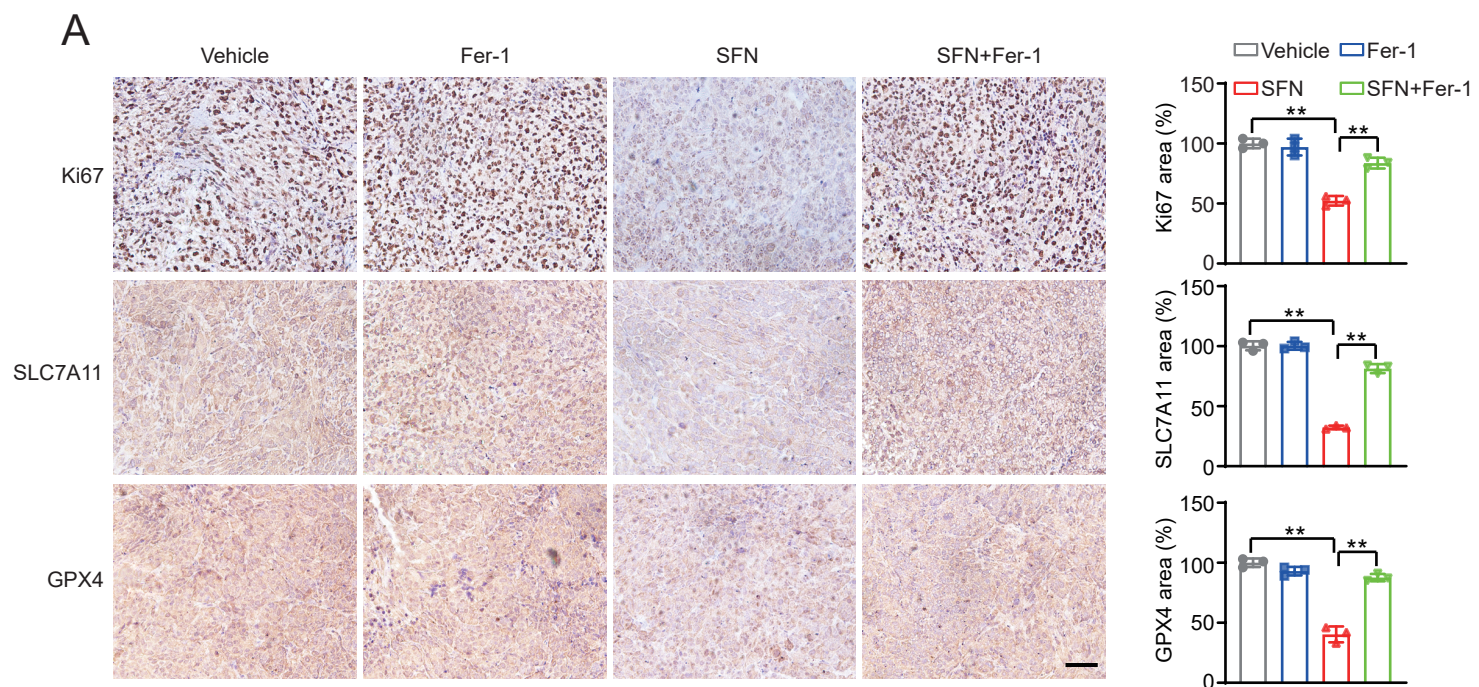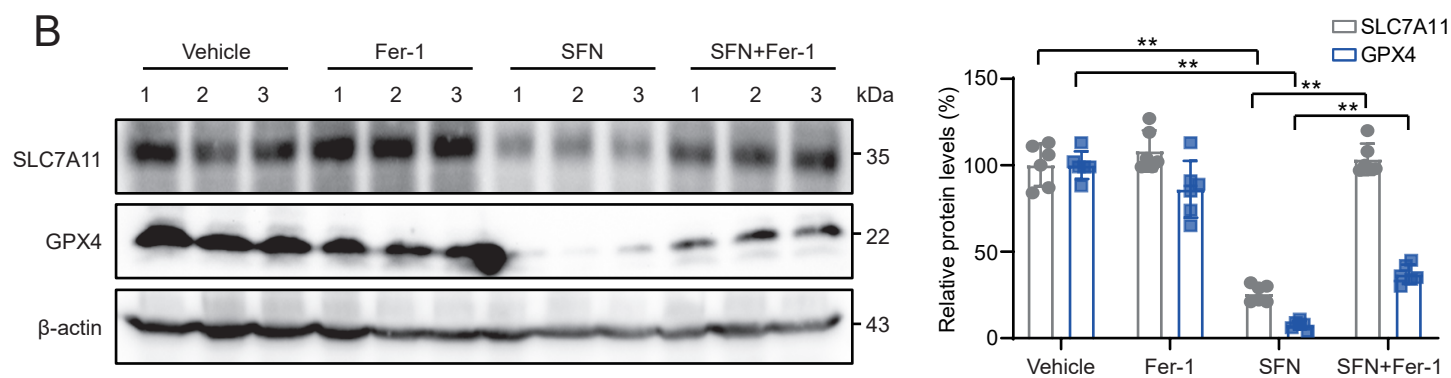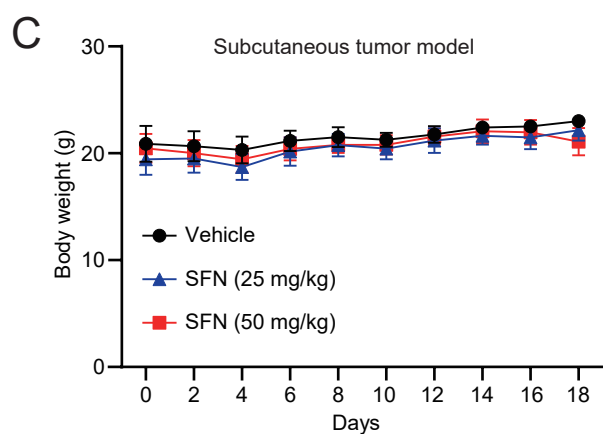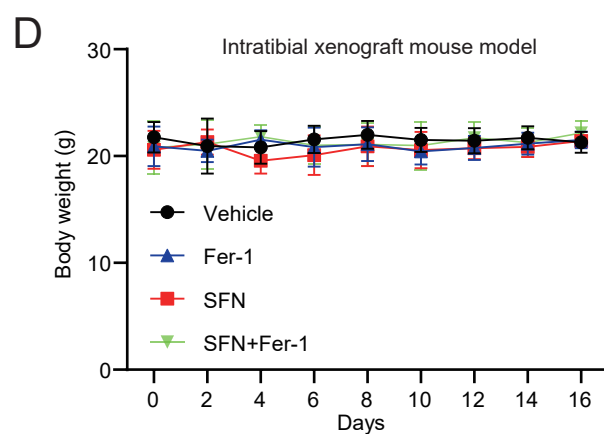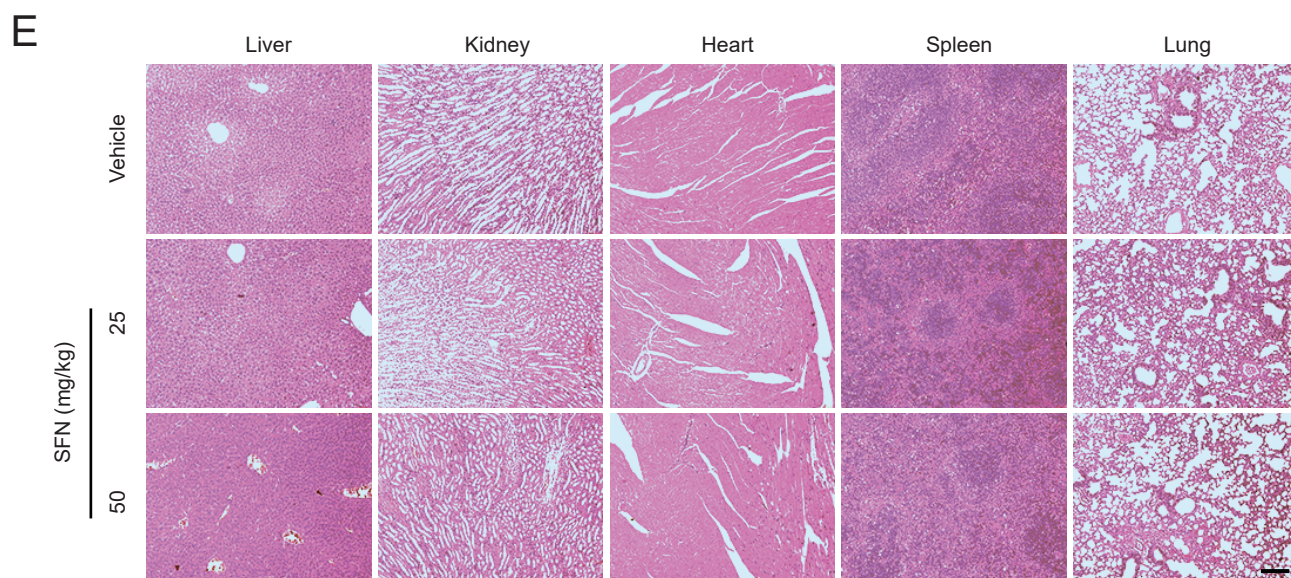

Supplement: Supplementary file 7 — Supplementary figure 7. Effect of SFN on OS xenograft in vivo. (A) IHC to determine the levels of Ki67, SLC7A11, and GPX4 in tumor tissues from intratibial xenograft mouse model. Scale bar: 200 μm. (B) The protein expression levels of SLC7A11 and GPX4 in tumor tissues intratibial xenograft mouse model. (C, D) The body weights of tumor bearing mice in subcutaneous (C) and intratibial (D) xenograft models were measured every other day. (E) H&E staining of liver, kidney, heart, spleen, and lung from animals treated with vehicle control or 25 or 50 mg/kg of SFN. Scale bar: 100 μm ∗P < 0.05, ∗∗P < 0.01. [file mmc7.pdf]
